# Anomalous Origin of the Right Coronary Artery From the First Septal Perforator

**DOI:** 10.7759/cureus.25784

**Published:** 2022-06-09

**Authors:** Masi Javeed, Rami Akel, Najam Javeed

**Affiliations:** 1 Internal Medicine, Hospital Corporation of America (HCA) Florida Bayonet Point Hospital, Hudson, USA; 2 Interventional Cardiology, Hospital Corporation of America (HCA) Florida Bayonet Point Hospital, Hudson, USA; 3 Interventional Cardiology, Hospital Corporation of America (HCA) Florida Trinity Hospital, Trinity, USA

**Keywords:** septal perforator, coronary artery angiography, "anomalous coronary artery", anomalous rca, right coronary artery (rca)

## Abstract

A 68-year-old white male presented to the clinic for chest pain and shortness of breath with exertion. Through coronary angiography, the patient was found to have an anomalous origin of the right coronary artery off the first septal perforator branch of the left anterior descending artery. The patient was treated with conservative medical therapy as symptoms had resolved, and the patient did not wish to undergo further procedures.

## Introduction

The incidence of all coronary artery anomalies in the general population is 1% [1]. An anomalous right coronary artery has an incidence of 0.26% [2]. The origin of an anomalous right coronary artery generally occurs from the left sinus of Valsalva, the posterior sinus of Valsalva, the ascending aorta, the pulmonary artery, the left main common artery, and the left circumflex artery [3]. Anomalous origin of the right coronary artery from the left anterior descending artery is still rare. Usually, it originates from the proximal or mid-portion of the left anterior descending artery; it also frequently originates distal to the first septal perforator and sometimes distal to the second septal perforator [4]. Less than 50 cases have been reported of the right coronary artery stemming from the left anterior descending artery [1-7]. In this report, we present an exceptionally rare case of an anomalous right coronary artery originating from the first septal perforator. 

## Case presentation

A 68-year-old white male with a past medical history, including peripheral arterial disease, carotid artery disease, hypertension, hyperlipidemia, alcohol use disorder, former tobacco use disorder, obesity, and testicular cancer status-post resection, presented to the clinic for four weeks of chest pain and shortness of breath with exertion. The patient’s cardiac medications included clopidogrel, lisinopril, hydrochlorothiazide, rosuvastatin, and ezetimibe. Initial vitals, physical examination, and labs were unremarkable. 

Electrocardiogram revealed nonspecific ST and T wave changes. Transthoracic echocardiogram showed an ejection fraction of 50%-55%, grade 1 diastolic dysfunction, mild mitral regurgitation, mild aortic regurgitation, mild tricuspid regurgitation, and mild pulmonary hypertension. A nuclear stress test was also ordered, which revealed a large, severe inferior fixed perfusion defect with partial reversibility. Upon discussion with the patient, the decision was made to proceed with cardiac catheterization or coronary angiography due to the partial, reversible perfusion defect.

**Figure 1:**
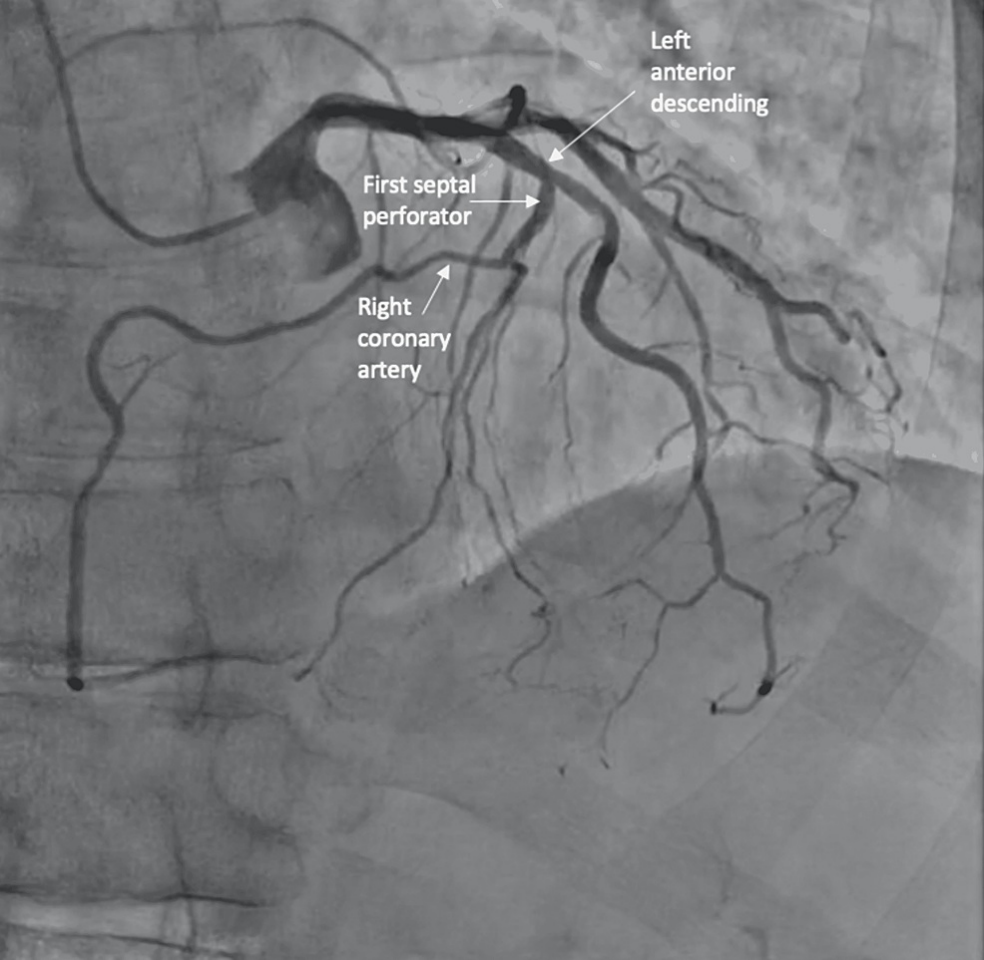
Coronary angiogram from the cranial and left angle oblique view demonstrated the anomalous origin of the right coronary artery from the first septal perforator.

**Figure 2:**
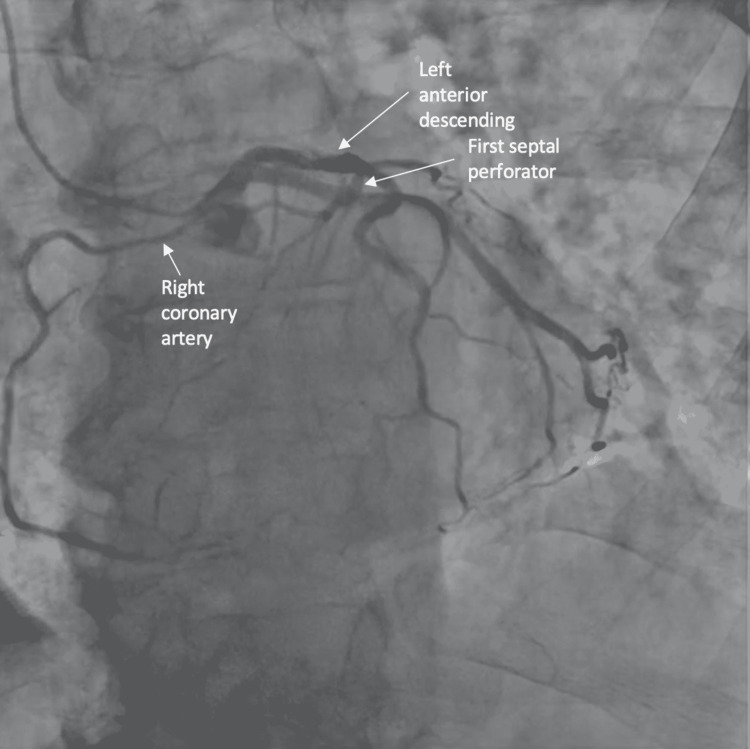
Coronary angiogram from the caudal and left angle oblique view demonstrated the anomalous origin of the right coronary artery from the first septal perforator.

**Figure 3:**
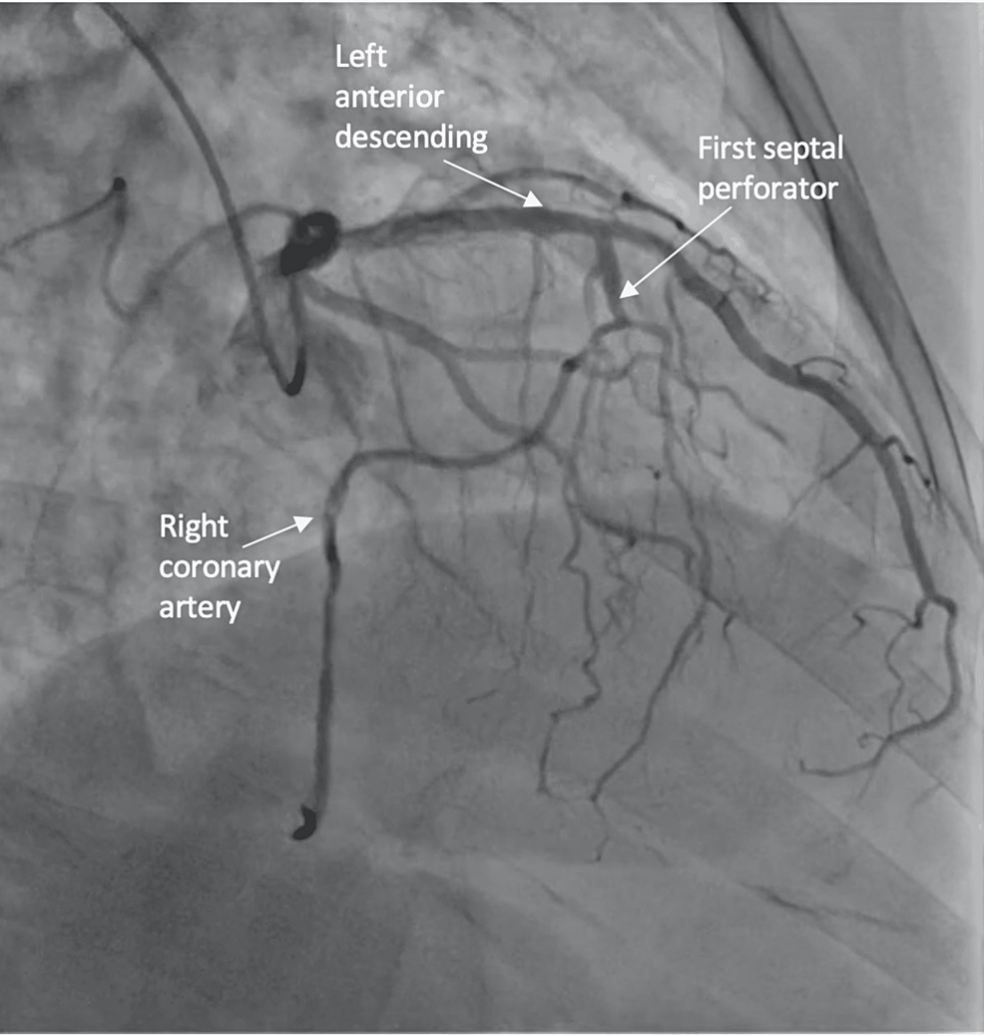
Coronary angiogram from the cranial and right-angle oblique projection demonstrated the anomalous origin of the right coronary artery from the first septal perforator.

Coronary angiography revealed an anomalous right coronary artery originating from the first septal perforator as well as a focal, eccentric, spontaneous coronary artery dissection in the mid-left anterior descending artery distally (Figures 1-3). 

A 3.0-millimeter x 12-millimeter Onyx drug-eluting stent was deployed in the mid-left anterior descending artery, adequately sealing the spontaneous coronary artery dissection. The patient tolerated the procedure well & no complications were noted. Dual-antiplatelet therapy was ordered for six months.

**Figure 4:**
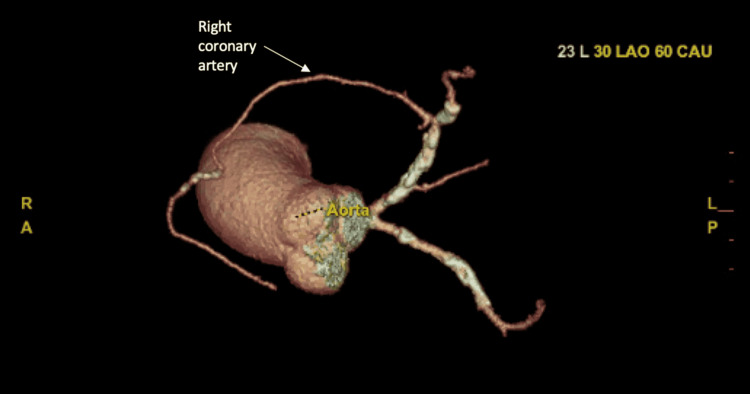
Three-dimensional coronary computed tomography angiography showed the anomalous right coronary artery.

**Figure 5:**
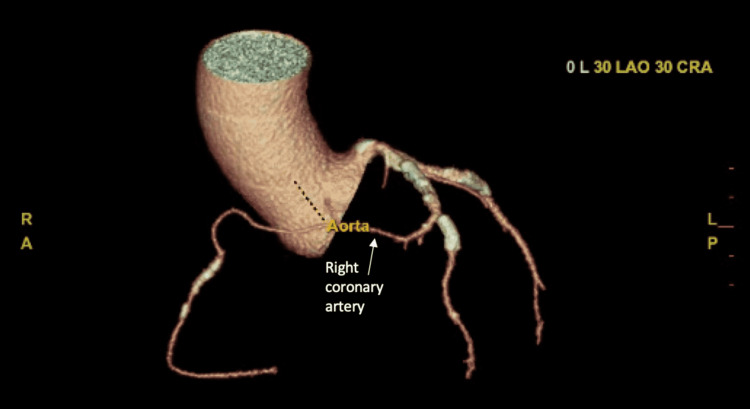
Three-dimensional coronary computed tomography angiography showed the anomalous right coronary artery.

Of special interest in the aforementioned procedure was the anomalous origin of the right coronary artery of the first septal perforator branch of the left anterior descending artery. This right coronary artery was also found to be dominant, diffusely less than 1 millimeter in luminal diameter, and with diffuse mild atherosclerosis. This artery was further evaluated by coronary computed tomography angiography revealing a non-malignant course of the anomalous right coronary artery (Figures 4, 5).

Due to the patient reporting resolution of symptoms, conservative medical therapy was pursued. The patient remained asymptomatic on follow-up a few weeks later.

## Discussion

In this case, we present an anomalous right coronary artery originating from the first septal perforator. To the best of our knowledge, only one other case of this has ever been reported [5]. 

Patients with coronary artery anomalies can be asymptomatic or experience angina, syncope, or even sudden cardiac death [6]. Some of the proposed mechanisms by which these symptoms occur include spasm of the anomalous artery, the acute angle of takeoff of the anomalous artery, the shape of the orifice from which the anomalous artery originates, and an intramural course of the anomalous artery [3]. The risk of myocardial ischemia or sudden cardiac death especially increases if the anomalous vessel has a malignant or inter-arterial course [7]. This is due to possible compression of the anomalous artery between the pulmonary and aortic trunks, particularly during or immediately after exercise [3]. 

Coronary angiography is the main diagnostic tool for coronary artery anomalies. Although this is the case, coronary computed tomography angiography and cardiac magnetic resonance imaging are both helpful as well. Coronary computed tomography angiography has high spatial resolution and, therefore, can provide high-quality images of both the proximal and distal coronary systems. Cardiac magnetic resonance imaging has lower spatial resolution and, therefore, the distal coronary system is not as well delineated. However, it may be superior in patients with congenital defects [3]. 

Treatment options for coronary artery anomalies include medical, percutaneous coronary intervention, or surgery. There have been multiple cases where percutaneous coronary intervention and cardiovascular surgery, including bypass grafting of the right coronary artery, have been performed for an anomalous right coronary artery originating from the left coronary system. However, the long-term benefits of these therapies have not yet been confirmed [3]. The management of coronary anomalies is still with no consensus and is controversial. Medical treatment is generally proposed in asymptomatic patients with no malignant subtype and in the absence of associated severe coronary artery disease or overt ischemia. Surgical treatment is generally proposed in symptomatic patients with a malignant coronary artery course and in the presence of severed coronary artery disease and overt ischemia. Percutaneous coronary intervention may be an alternative to surgery, especially in patients with high surgical risk and complex coronary anatomy [7].

## Conclusions

We conclude that there are very few cases of an anomalous right coronary artery originating from the left coronary system. Furthermore, to the best of our knowledge, this is only the second reported case of an anomalous right coronary artery stemming from the first septal perforator branch of the left anterior descending artery.

Coronary angiography is the gold standard for diagnosis. However, cardiac magnetic resonance imaging and coronary computed tomography angiography are also useful. Treatment for coronary artery anomalies usually consists of medical therapy if the patient is asymptomatic or the artery is not malignant. Surgery as well as percutaneous coronary intervention should be considered otherwise.

## References

[1] Jammula P, Gupta R, Uretsky BF (2005). Images in cardiology: Anomalous origin of the right coronary artery from the left anterior descending artery. Heart.

[2] Canbay A, Ozcan O, Vural M, Diker E (2008). A rare coronary artery anomaly: anomalous right coronary artery arising from the left anterior descending artery. Int J Cardiol.

[3] Yurtdas M, Gulen O (2012). Anomalous origin of the right coronary artery from the left anterior descending artery: review of the literature. Cardiology journal.

[4] Gholoobi A (2016). Anomalous origin of the right coronary artery from the midportion of the left anterior descending artery: a rare coronary anomaly. J Tehran Heart Cent.

[5] Meyers DG, McManus BM, McCall D, Walsh RA, Quaife MA (1984). Single coronary artery with the right coronary artery arising from the first septal perforator. Cathet Cardiovasc Diagn.

[6] Salih M, Abdel-Hafez O, Ibrahim R, Halabi AR, Aloka F (2020). A single coronary artery anomaly: Right coronary artery as a branch from the left anterior descending artery. Cureus.

[7] Jrad M, Ammar A, Affes M (2016). Anomalous origin of right coronary artery from the left anterior descending artery. Angiology: Open access.

